# Improved detection of sentinel lymph node metastases allows reliable intraoperative identification of patients with extended axillary lymph node involvement in early breast cancer

**DOI:** 10.1007/s10585-020-10065-9

**Published:** 2020-11-29

**Authors:** Cristina L. Cotarelo, Annemarie Zschöck-Manus, Marcus Schmidt, Arno Schad, Christoph Brochhausen, Charles James Kirkpatrick, Sonja Thaler

**Affiliations:** 1 Institute of Pathology, Heinrich Heine University and University Hospital of Duesseldorf, Moorenstr. 5, D-40225 Düsseldorf, Germany; 2 Institute of Pathology, University Medical Center of Johannes Gutenberg University, Mainz, Germany; 3 Department of Gynecology and Obstetrics, University Medical Center of Johannes Gutenberg University, Mainz, Germany; 4 Institute of Pathology, University Regensburg, Regensburg, Germany; 5 European Centre for Angioscience (ECAS), Medical Faculty Mannheim, University of Heidelberg, Mannheim, Germany

**Keywords:** Sentinel lymph node, Metastases, Touch imprint cytology, Frozen section

## Abstract

An improved procedure that allows accurate detection of negative sentinel lymph node (SLN) and of SLN macrometastases during surgery would be highly desirable in order to protect patients from further surgery and to avoid unnecessary costs. We evaluated the accuracy of an intraoperative procedure that combines touch imprint cytology (TIC) and subsequent frozen section (FS) analysis. 2276 SLNs from 1072 patients with clinical node-negative early breast cancer were evaluated during surgery using TIC. Only cytologically-positive SLN were subsequently analysed with a single FS, preserving cytologically-negative SLN for the final postoperative histological diagnosis. Sensitivity, specificity and the accuracy of this approach were analysed by comparing the results from intra- and postoperative SLN and axillary node evaluation. This intraoperative method displayed 100% specificity for SLN metastases and was significantly more sensitive for prognostically relevant macrometastases (85%) than for micrometastases (10%). Sensitivity was highest for patients with two or more positive LNs (96%) than for those with only one (72%). 98% of the patients with final pN2a-pN3a were already identified during surgery. Patients who received primary axillary lymph node dissection had significantly more frequent metastases in further LNs (44.6%). Sensitivity was highest for patients with luminal-B, HER2+ and triple negative breast cancer and for any subtype if Ki-67 > 40%. TIC and subsequent FS of cytologically-positive SLNs is highly reliable for detection of SLN macrometastases, and allows accurate identification of patients with a high risk of extended axillary involvement during surgery, as well as accurate histological diagnosis of negative SLN.

## Introduction

In most cases of breast cancer sentinel lymph node (SLN) metastases is determined by standard postoperative evaluation [1–3]. However, some patients with postoperatively-diagnosed SLN metastases are forced to undergo a second surgical intervention for complete axillary lymph node dissection (ALND). The main advantage of an intraoperative SLN assessment is therefore that metastases can be diagnosed and removed in a single surgical procedure. Nevertheless, there are several drawbacks that raise doubts about its use. These doubts include principal concerns about accuracy but also the current view that not all patients with positive SLN should undergo a complete ALND [4, 5].

The published guidelines to process SLN in breast cancer do not define a standard assessment procedure for intraoperative evaluation [2, 3]. In the literature different techniques are described for potential intraoperative evaluation of SLN such as touch imprint cytology (TIC), frozen section (FS) analysis, rapid cytokeratin immunostaining or combinations of these methods [6, 7]. The use of intraoperative assessment can be beneficial for those patients with a histologically positive nodal status, but an accurate negative intraoperative evaluation for patients without metastases is still needed. It is known that intraoperative assessment can compromise the final diagnosis of SLN. FS has several disadvantages, including the loss of tissue through the sectioning process as well as distortion of the tissue architecture [8, 9]. Cytological techniques such as TIC can prevent these negative sequelae. However, although the cut surface of the SLN is preserved, the disadvantage of this cytological technique is its low accuracy and its low specificity to detect SLN metastases [10, 11].

Furthermore, it has been proposed that axillary dissection could be avoided in selected patients with positive SLN who meet criteria that include T1 or T2 primary lesions, one or two positive axillary SLN without extra-capsular infiltration, and who plan to undergo breast-conserving surgery followed by conventionally fractionated whole-breast radiotherapy [2–5]. This newly postulated management regimen raises the question of whether the histological diagnosis of SLN might be necessary directly during surgery for patients with early breast cancer.

However, many patients with postoperatively-diagnosed SLN metastases currently receive a second operation for ALND. Therefore, a method for the detection of SLN metastases that allows reliable intraoperative prediction of those patients with the need for ALND in early breast cancer is highly desirable in order to protect patients from undergoing a second surgical procedure and to avoid unnecessary costs.

A previously reported study described a major improvement of intraoperative SLN diagnostic by the use of TIC and subsequent FS of all SLNs, giving 86.2% sensitivity, 98.9% specificity and 96% accuracy, when evaluating the data per case [10]. In our study, we modified this procedure by using TIC as a screening system to detect metastatic tumor cells in a first step. In contrast with others, we only prepared FS for further diagnosis if SLNs display cytologically detectable tumor cells. In these cases, a single frozen section was prepared on the suspect surface of the SLN and used for intraoperative diagnosis as a second step. Since it is known that essentially all micro- and macrometastases can only be detected by step sectioning of the entire paraffin-embedded SLN [12–14], the main advantage of this modified procedure is that tissue from cytologically tumor cell negative SLNs is completely preserved for subsequent postoperative histological analysis and thus allows an accurate intra- and postoperative evaluation of the SLNs.

In our study, we included all 1072 clinical node-negative early breast cancer cases that were sent to the Institute of Pathology at the University Medical Center in Mainz between 2010 and 2013. All patients underwent breast surgery and SLN dissection. In total, 2276 SLNs were evaluated during surgery. We tested the value of our modified intraoperative procedure by evaluating the sensitivity, specificity and the accuracy to detect SLN metastases. We also investigated whether the outcome of this procedure for patients depends on the molecular breast cancer subtype, on the tumor's proliferation index or on the number of positive LN respectively.

## Materials and methods

### Patient data and patient sample collection

1072 patients with primary early breast cancer and negative preoperative axillary ultrasound were included in this study. Patients received an SLN resection and modified radical mastectomy or breast-conserving therapy. All patients had pathological evaluation carried out at the Institute of Pathology of the University Medical Centre of the Johannes Gutenberg University of Mainz, Germany between January 2010 and December 2013. This period was selected for our retrospective study because during this period all patients with an SLN biopsy underwent intraoperative evaluation. No patients with a clinically negative node breast cancer were selected for a postoperative histological evaluation alone. 2276 SLNs (median 2.1; range 1–8 nodes per patient) were processed for intraoperative evaluation.

Clinico-pathological data were compiled, and included patient age, histological tumor type according to the WHO classification of breast tumors [15], histological grading of the tumor according to the Nottingham histological score system [16], TNM classification according to the 7th edition from 2009, estrogen receptor status (ER), progesterone receptor status (PR), HER2-neu status (HER2) and proliferation index (Ki-67). The original pathology reports were used, but all cases were re-evaluated.

715 of the 1072 cases were able to be classified according to the St. Gallen International Breast Cancer Conference from 2013 with its suggested definition of intrinsic subtypes of breast cancer: luminal-A (ER+, PR+, Ki-67 low (</= 20%) and HER2-), luminal-B (ER+, PR low/-, Ki-67 high and HER2-), luminal-HER2 (ER+, PR low/- and HER2+), HER2+ (ER-, PR- and HER2+) and triple negative (ER-, PR- and HER2-) [17]. The luminal-A and luminal-B could not be differentiated in 357 cases because the Ki-67 indices from these primary breast tumors were not available.

### Intraoperative preparation and evaluation of SLNs

**Fig. 1:**
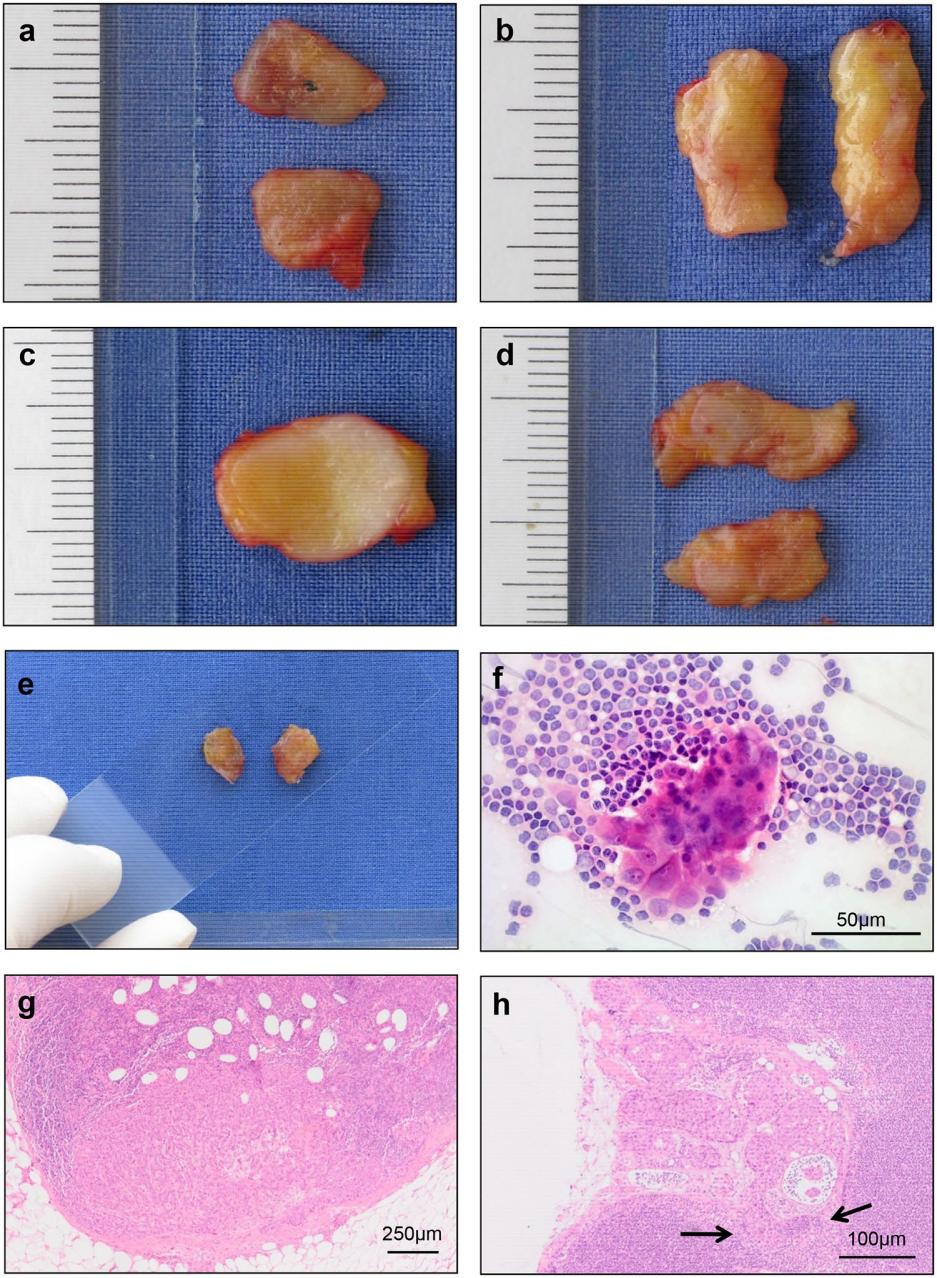
**P**reparation and evaluation of SLNs. **a**, **b** Initially, gross evaluation of the tissue was performed. Some SLNs were not grossly suspicious. **c**, **d** Other SLNs were suspicious on gross examination. **e** Subsequently, touch preparation cytology was performed from each surface of the bisected SLNs by pressing the surfaces onto a slide. The slides with the imprints were fixed and stained with (H&E). **f** If tumor cells were cytologically identified the corresponding section of the SLN was used for intraoperative diagnosis. Only one frozen section was prepared and stained with H&E. **g** The final intraoperative positive diagnosis was based on a SLN with a focus of metastatic carcinoma in the frozen section. **h** Step sectioning of SLNs after formalin fixation and paraffin-embedding offers optimal conditions for the detection of metastases. This procedure allows the evaluation of the entire subcapsular sinus, the location within LNs where metastases first start to grow. Metastatic tumor cells are indicated by arrows

Firstly, gross evaluation of the tissue was performed to determine the number of nodes. The SLNs were bisected if the width was < 5 mm or sliced into 2 mm thick sections if the width was > 5 mm (Fig. 1a–d). Subsequently, touch preparation cytology was performed from each surface of each section (Fig. 1e). The slides with the imprints were fixed with M-FIX^TM^ spray fixative (Merck, Darmstadt, Germany) and stained with hematoxylin and eosin (H&E), using standard laboratory procedures. Only for those nodes in which tumor cells were cytologically identified was the corresponding section of the SLN frozen for intraoperative diagnosis (Fig. 1f). Only one frozen section was obtained and stained with H&E. The final intraoperative positive diagnosis was based on a SLN with a focus of metastatic carcinoma in the frozen tissue (Fig. 1g).

All patients with a final intraoperative positive SLN received an immediate ALND.

### Postoperative processing of SLNs

**Fig. 2:**
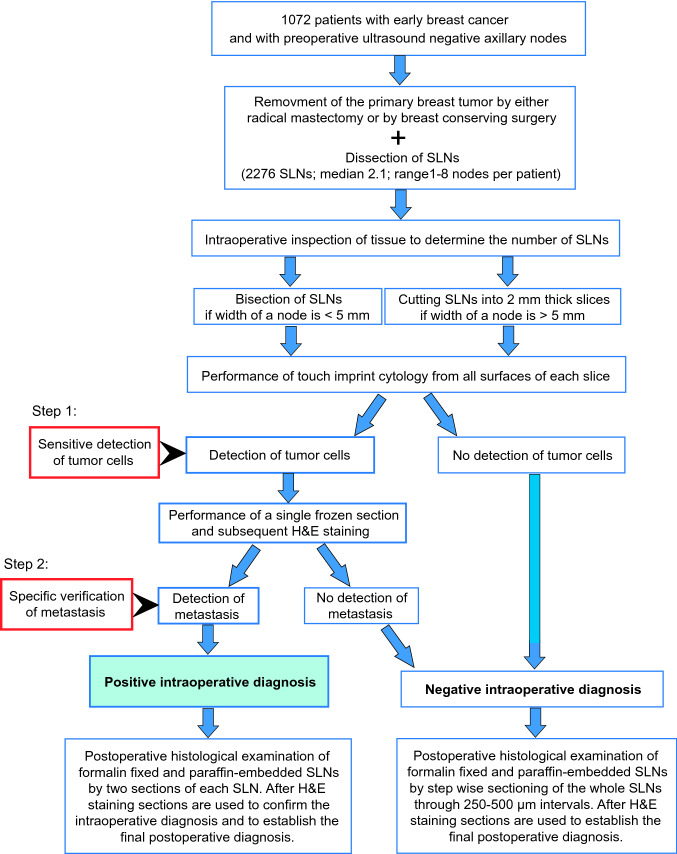
Schematic overview of the procedure used for this study. We used TIC as a screening system to detect SLN metastasis in a first step. We only prepared FS for further investigation if SLNs display cytologically detectible tumor cells. In these cases, only a single frozen section was prepared from the suspect surface of the SLN and used for intraoperative diagnosis as a second step

The residual tissue from all SLNs was fixed in 4% neutral-buffered formalin. Two additional sections (4–6 µm thick) were cut from each face of the intraoperative positive SLN segment for final histology after H&E staining of the samples. For all intraoperative negative SLN, the residual tissue was completely sectioned using 250 µm distance between two sections, yielding approximately 8 sections per specimen. Step sectioning of SLNs after formalin fixation and paraffin-embedding offers optimal conditions for the detection of metastases. This procedure allows the evaluation of the entire subcapsular sinus, the location within a lymph node where metastases first start to grow. Metastatic tumor cells are indicated by arrows (Fig 1h). [1, 12]. A schematic overview of the intra- and postoperative procedure is given in Fig. 2.

### Definition of final diagnosis (nodal status)

According to established recommendations, immunohistochemistry (IHC) of SLNs was not automatically performed [18]. Immunohistological detection of cytokeratin was used only when the presence of micrometastases was suspected, but not certain, or when a suspicious focus mimicked benign histiocytes in SLNs from patients with known invasive lobular carcinoma. Micrometastases, macrometastases and isolated tumor cells were documented according to the TNM-classification of malignant Tumors (7th Edition, 2009).

### Evaluation of false negative SLNs

The cytological specimens, frozen section and paraffin-embedded slides from false negative SLNs were reviewed independently by two pathologists to determine the reason for discrepancy between the result of intraoperative evaluation and the final postoperative diagnosis. We classified the underlying cause of the discrepancy as method error (ME), sampling error (SE) or misinterpretation (MI). ME was defined as the finding of a metastasis in the first level of the cut surface of the SLN by negative intraoperative evaluation; SE was defined as a finding of a metastasis in a deeper level of SLN by negative intraoperative evaluation; and MI was defined as misinterpretation of a focus of metastatic carcinoma on the frozen section by cytologically positive node.

### Calculation of sensitivity, specificity, the positive-, negative predictive value and accuracy

Sensitivity = TP/(TP+FN); Specificity = TN/(TN+FP); PPV, positive predictive value = TP/(TP+FP); NPV, negative predictive value = TN/(TN+FN) and accuracy (TN+TP)/total number of cases).

### Statistical evaluation

Statistical significance was evaluated by the using the Fisher´s exact test. A p value of < 0.001 was considered to be statistically significant [19].

## Results

### Patient data and sample collection

SLNs from 1072 patients were evaluated. All patients had no previous history of malignant disease. Thirteen patients had concurrent bilateral breast cancer. The ages ranged from 28 to 94 years, with a median of 61.3 years. The division of this patient collective into the main molecular breast cancer subtypes and other clinico-pathological data is shown in Table 1. 145/1072 (13.5%) patients had one or more criteria of ≥ T3 primary tumor or ≥ 3 positive LN or underwent a mastectomy.

**Table 1:** Clinico-pathological data of the patients

Histologic tumor type (n = 1072)		Grading (n=1072)		pT-stage (n=1072)		Molecular subtype (n=1072)	
Invasive ductal carcinoma	798 (74.44%)	G1	299 (27.89%)	pT1a-c	647 (60.35%)	**luminal**	854 (79.6%)
Invasive lobular carcinoma	148 (13.80%)	G2	505 (47.10%)	pT2	371 (34.60%)	A	431
Invasive mucinous carcinoma	27 (2.5%)	G3	268 (25%)	pT3	51 (4.75%)	B	66
Other types of invasive carcinoma	99 (9.2%)			pT4b	3 (0.27%)	A/B unidentified	357
						HER2	101 (9.4%)
						luminal	63
						** non luminal **	38
						Triple negative	117 (10.9%)

### Comparison between intra- and postoperatively diagnosed macro- and micrometastases

Final postoperative histopathological assessment of all 1072 patients revealed 287 (26.8%) patients with axillary lymph node metastases. 269 of these 287 patients had one or more positive SLNs (25.1% of the 1072 patient cohort). 219 patients with SLN metastases had SLN macrometastases (20.4% of the 1072 patient cohort) whereas 50 (4.7% of the 1072 patient cohort) patients displayed SLN micrometastases (Table 2). 18 of the 287 patients with axillary lymph node metastases (1.6% of the 1072 patient cohort) were classified as SLN-negative in the intraoperative and postoperative histopathological assessment, but an intraoperatively suspicious non-sentinel axillary lymph node proved to have macrometastasis in the histopathological assessment. In 1.6% of cases, the SLN identification was false. This rate is consistent with previously published analyses [20].

**Table 2:** Comparison between intra- and postoperative diagnosed macro- and micrometastases

	Total	Micro- metastases in SLN	Final nodal stage			Macro- metastases in SLN	Final nodal stage		
			pN1mi	pN1a	pN2a		pN1a	pN2a	PN3a
Positive SLNs (N=1072)	269 (25.1%)	50 (4.7%)	46 (4.3%)	3 (0.3%)	1 (0.1%)	219 (20.4%)	159 (15.3%)	40 (3.7%)	20 (1.8%)
Positive intraoperatively diagnosed SLNs	191 (71%)	5 (10%)	3 (6.5%)	2 (66.7%)		186 (84.9%)	126 (79.2%)	40 (100%)	20 (100%)
Negative intraoperatively diagnosed SLNs	78 (29.0%)	45 (90%)	43 (93.5%)	1 (33.3%)	1 (100%)	33 (15.1%)	33 (20.8%)		

In the final postoperative assessment, 46 (17%) patients had only SLN micrometastasis and 94 (35%) patients had only one positive lymph node (LN), whereas 50 (18.5%) patients had two positive LNs and 79 (29.5%) patients had three or more positive LNs. The final nodal stage of all patients with positive SLNs (N = 269) and the comparison between the intraoperative diagnosis and the final nodal stage in patients who had macro- or micrometastases within SLNs are shown in Table 2. 100% of the patients who demonstrated a macrometastasis within the SLN and final pN2a-pN3a were already identified during surgery and underwent primary ALND. Only one patient with a SLN micrometastasis and final pN2a staging underwent a secondary ALND.

### Differences between intra- and postoperative diagnoses in relation to sensitivity, specificity, PPV, NPV and accuracy in false negative cases

78 (7.2%) of all intraoperatively evaluated cases were classified as false-negatives (FN). 45 (4.2%) of them were micrometastases, whereas only 33 (3.1%) cases were macrometastases. The sensitivity of the method for all metastases (micrometastases and macrometastases) was 71%, the specificity and the positive predictive value (PPV) were 100%, while negative predictive value (NPV) was 91.1%. The accuracy was 92.7%. The sensitivity, specificity, PPV, NPV and the accuracy of the method for the SLN macrometastases and for the micrometastases are shown in Table 3. These results indicate that our combined intraoperative evaluation of SLN is significantly more sensitive for macrometastases (85%) than for micrometastases (10%) (p value < 0.001).

**Table 3:** Differences between intra- and postoperative diagnoses in relation to sensitivity, specificity, PPV, NPV and accuracy of false negative cases

	TP	FN	TN	FP	Sensitivity (%)	Specificity (%)	PPV (%)	NPV (%)	Accuracy (%)
Metastasis	191	78	803	0	71	100	100	91.1	92.7
Micrometastases	5	45	1022	0	10	100	100	95.8	95.8
Macrometastases	186	33	853	0	85	100	100	96.3	96.9

### Reason for discrepancies between intra- and postoperative diagnosis of false negative SLN metastases

The majority of the 78 false-negative cases were micrometastases (45 cases, 58%). The majority of the discrepancies between the cases (55 cases, 70.5%) were classified as sampling error. In these cases, metastatic tumor cells were found postoperatively in deeper sections of SLNs. 34 (61.8%) out of these 55 cases were micrometastases and only 21 (38.2%) cases were macrometastases. In 22 (28.2%) cases the discrepancies were classified as method error. The metastatic tumor cells were found postoperatively in the upper sections of SLNs. Only one case with a micrometastasis was classified as misinterpretation. This case displayed a metastatic focus that was not recognized on the original frozen section. The re-evaluation of all 78 cases found no misinterpretation of foci of metastatic carcinoma either in the original frozen section or the final postoperative diagnosis.

### Additional FS of cytologically negative SLNs fails to reduce the number of false negative cases

In order to evaluate the usefulness of additional FS in cytologically negative SLN we performed an additional frozen section on 197 cases with negative TIC. This approach did not increase the negative predictive value for metastases (83%), micrometastases (89%) or macrometastases (94%) in comparison to the NPV for metastases (91%), micrometastases (96%) and macrometastases (96%) of the described method. The relation of false negatively diagnosed SLNs between TIC alone or TIC combined with FS, the final diagnosis of SLNs and the explanation for the discrepancy between intra- and postoperatively diagnosed SLN metastases are shown in Table 4.

**Table 4:** Differences of false negative cases (N=78) between imprint cytology alone or imprint cytology with frozen section

	Total	TP	TN	FN	Micro- metastases in SLN	Reason for discrepancy			Final nodal stage			Macro-metastases in SLN	Reason for discrepancy		Final nodal stage
						MI	ME	SE	pN1mi	pN1a	pN2a		ME	SE	pN1a
TIC neg.	678		638	**40**	22		7	15	20	1	1	18	8	10	18
TIC neg./FS neg.	197		165	**32**	21		2	19	21			11	3	8	11
TIC pos./FS neg.	5			**5**	1		1		1			4	1	3	4
TIC pos./FS pos.	192	191		**1**	1	1			1						

### The sensitivity of our workflow for detecting SLN metastases varies according to the breast cancer molecular subtype and the proliferation index, but the differences are not statistically significant

The sensitivity of our intraoperative method for the detection of metastases within SLNs varies when different molecular breast cancer subtypes are compared. We observed that the sensitivity was highest for patients with luminal-B (92%), HER2 positive (91%) or triple negative tumors (91%), and was lowest for patients with luminal-A breast cancer (79.5%). However, the differences were not statistically significant. Similar observations were made through assessing the proliferation indices of tumors. Thus, the sensitivity was highest for patients with a proliferation index of more than 40% (91%) and lowest for patients with a proliferation index of 20% or less (80%).

The sensitivity, specificity, PPV, NPV and accuracy of the intraoperative diagnos is of SLN metastases with respect to the molecular subtype of breast cancer as well as the proliferation index are shown in Table 5.

**Table 5:** The sensitivity of our workflow for detecting SLN metastases varies according to the breast cancer molecular subtype and the proliferation index, but the differences are not statistically

	Total	TP	FN	TN	FP	Sensitivity (%)	Specificity (%)	PPV (%)	NPV (%)	Accuracy (%)
luminal A type	431	66	17	348	0	79.5	100	100	95.3	96
luminal B type	66	11	1	53	0	92	100	100	98.1	97
luminal HER2 type	63	15	2	46	0	88	100	100	95.8	96.8
triple negative type	117	21	2	94	0	91	100	100	97.9	98
HER2+ type	38	8	1	29	0	91	100	100	96,7	97
< 20% Ki-67	473	73	18	381	0	80	100	100	95.5	96
>20% Ki-67	156	29	5	121	0	85.3	100	100	96.0	96
>40% Ki-67	103	21	2	80	0	91	100	100	97.5	98

### The sensitivity of our workflow for detecting SLN metastases depends on the total number of axillary LN metastases but not on the number of intraoperatively evaluated SLNs

The present study also shows that the chosen intraoperative procedure to diagnose SLN metastases is significantly more sensitive for patients with three or more positive LN (98.6%) than for those patients with only one positive LN (68%) (p < 0.001) (Table 6). Furthermore, patients with an intraoperative positive SLN who received immediate ALND showed additional non-SLN metastases in 44.6% of cases (84 of 191). Patients with a positive SLN in standard postoperative assessment of SLNs who received a secondary ALND instead had additional non-SLN metastases in only 13.2% of cases (5 of 38) and were therefore significantly less frequent than those having immediate ALND (p < 0.001). 60 out of 61 patients with a final lymph node stage greater than pN1a were identified through intraoperative evaluation and obtained an immediate ALND. The sensitivity of our workflow for detecting SLN metastases does not depend on the number of intraoperatively evaluated SLNs (Table 7).

**Table 6:** The sensitivity of our workflow for detecting SLN metastases depends on the number of axillary LN metastases

n = 269	SLN	Positive	TP	FN	TN	FP	Sensitivity (%)	Specifity (%)	PPV (%)	NPV (%)	Accuracy (%)
1 positve axillary LN (n = 136)	Metastasis	136	67	69	936	0	49.2	100	100	92	93.5
	Micrometastases	42	3	39	1030	0	7.1	100	100	95.3	96.3
	Macrometastases	94	64	30	978	0	**68**	100	100	96.3	97.2
2 positve axillary LN (n =54)	Metastasis	54	48	6	1018	0	92.3	100	100	99.2	99.4
	Micrometastases	4	0	4	1068	0	0	100	100	99.5	99.6
	Macrometastases	50	48	2	1022	0	**96**	100	100	99.7	99.8
3 or > positive LN (n = 79)	Metastasis	79	76	3	993	0	96.2	100	100	99.6	99.7
	Micrometastases	3	1	2	1069	0	33	100	100	99.7	99.8
	Macrometastases	76	75	1	996	0	**98.6**	100	100	99.8	99.9

**Table 7:** The sensitivity of our workflow for detecting SLN metastases does not depend on the number of intraoperatively evaluated SLNs

n = 1072		Positive	TP	FN	TN	FP	Sensitivity (%)	Specifity (%)	PPV (%)	NPV (%)	Accuracy (%)
1 SLN (n = 464)	Metastasis	96	68	28	368	0	70.8	100	100	92.9	93.9
	Micrometastases	18	1	17	446	0	5.5	100	100	96.3	96.3
	Macrometastases	78	67	11	386	0	**85.8**	100	100	97.2	97.6
2 SLN (n = 300)	Metastasis	78	58	20	222	0	74.3	100	100	91.7	93.3
	Micrometastases	14	3	11	286	0	21	100	100	96.2	96.3
	Macrometastases	64	54	10	236	0	**84.3**	100	100	95.9	96.6
3 SLN (n = 163)	Metastasis	49	33	16	114	0	67.3	100	100	87.69	90.1
	Micrometastases	11	0	11	152	0	0	100	100	93.2	93.2
	Macrometastases	38	33	5	125	0	**86.8**	100	100	96.1	96.9
4 SLN (n = 79)	Metastasis	28	20	8	51	0	71.4	100	100	86.4	89.9
	Micrometastases	4	0	4	75	0	0	100	100	94.9	94.9
	Macrometastases	24	20	4	55	0	**83.3**	100	100	93.2	94.9
> 4 SLN (n = 66)	Metastasis	18	13	5	48	0	72.2	100	100	90.5	92.4
	Micrometastases	3	1	2	63	0	33.3	100	100	96.9	96.96
	Macrometastases	15	12	3	51	0	**80**	100	100	94.4	95.4

## Discussion

In patients undergoing a SLN biopsy, combined or non-combined usage of TIC and FS are the most frequently used techniques for intraoperative SLN evaluation [21] . The main advantage of TIC is that it is rapid, cost-effective and preserves tissue for subsequent analyses. However, the disadvantages are low accuracy and low specificity for detecting SLN metastases [10, 11] . When compared with the results from final postoperative histopathological assessment, the specificity of FS is close to 100%. However, this technique is expensive, time-consuming and has several diagnostic disadvantages, including the sacrifice of tissue as a result of the sectioning process as well as distortion of the tissue architecture [7–9] . Nagashima et al. found a major improvement in diagnostic performance by combining the two techniques, giving 86.2% sensitivity, 98.9% specificity and 96% accuracy, when evaluating the data per case [10] .

In the present study we combined both methods, but we used TIC as a screening system to decide which SLN is possibly positive and should be further investigated by frozen section (Fig. 2). For these cases, only a single frozen section was performed on the suspect surface of the SLN in order to reduce tissue loss. The purpose of this altered two-step procedure was to establish a method that allows detection of tumor cells within SLNs in an accelerated, but maximally sensitive way. Only those SLNs that contained tumor cells as evidenced by TIC were screened by FS to allow verification of the TIC result. This additional FS step proves in a specific way that the SLNs indeed contain metastases. This combined intraoperative approach using TIC and FS has a specificity of 100%. In this respect, no patient without positive nodal status underwent an ALND in our study. Additionally, this intraoperative procedure preserves the tissue from intraoperative negative SLN for the final histological diagnosis, which is advantageous, since it is known that micro- and macrometastases can only be efficiently detected by analysing step sections from SLN blocks after formalin fixation and paraffin embedding [12, 13] . This procedure optimizes the detection of metastases by enabling the evaluation of the entire subcapsular sinus, the location where metastatic tumor cells first start to grow. It is therefore the typical site where small lymph node metastases are found (Fig 1h) [12, 13] . We achieved with our intraoperative approach 100% specificity for all metastases and 85% sensitivity for macrometastases (Table 3). This result is comparable with findings of previously reported studies [10, 11] . Thus, through the combination of both techniques, there are the advantages of 100% specificity of the FS without loss of sensitivity, and the preservation of tissue for final histological and immunohistochemical assessment. Furthermore, other advantages are gained by this combined approach, namely a relatively quick procedure and a cost-effective evaluation of SLN, which is in accordance with previous results in the literature [22] . Regarding eligibility for ALND under current guidelines 13.5% of the patients in this study would have been candidates for ALND based on Z0011 criteria [5] . This Information clarifies the potential impact of this intra-operative pathologic evaluation. Based on these findings, we conclude that our workflow of intraoperative SLN evaluation represents a new and clinically useful approach.

Similar to the findings of previous studies our results indicate that the majority of our false negatively diagnosed SLNs (70.5% of all FN) are caused through sampling error [23, 24] . This means that the highest risk of false negative results is given in those cases with small tumor burden (patients with micrometastases or with only one positive axillary lymph node (Tables 2 and 6)) and their metastatic tumor cells can only be detected by use of the step-section technique from SLN blocks after formalin fixation and paraffin embedding. Furthermore, we found that only 13.15% of patients with a false-negative intraoperative SLN had any additional positive non-SLN, while 44.6% of patients with a positive intraoperative SLN had additional positive non-SLN [23] , and that the sensitivity of our intraoperative combined method for patients with 2 or more positive LN was excellent (96%) (Table 6).

These findings support the concept that the most of the patients with an intraoperative false negative SLN had limited axillary involvement. These patients clearly carry a good prognosis, and studies show that dissecting the axilla in the presence of positive SLNs in selected patients with limited SLN metastases does not achieve any advantage for loco-regional control or survival, if the patients are treated with breast conservation surgery and whole breast irradiation [4, 5, 25] .

The more extensive degree of axillary LN involvement in patients who had immediate ALND after a positive intraoperative SLN has been published previously [26] . In contrast to that study, in our patient collective the overall representation of larger tumors (5.0%) and triple negative breast cancer (10.9%) was very low. Thus, greater representation of patients with larger tumors and triple negative breast cancer cannot explain a higher degree of axillary LN involvement in patients who had immediate ALND after a positive SLN was diagnosed intraoperatively. Additionally, we found that our intraoperative approach was highly sensitive for the detection of metastases in patients with triple negative breast cancer (91%) (Table 5) and patients with more than three positive LN (96.2%) (Table 6). A possible explanation for the higher frequency of additional non-SLN metastases and a higher representation of ER-negative tumors in those patients who underwent an immediate ALND is the higher sensitivity of the intraoperative evaluation, which we observed especially for those categories.

Questions regarding the clinical relevance of ALND in patients with SLN metastases are discussed controversially. Two prospective randomized clinical trials examined the omission of complete ALND in SLN-positive patients. These studies provided evidence that dissecting the axilla in the presence of positive SLNs does not achieve any advantage for loco-regional control or survival. This excellent local control without any change of prognosis by omitting ALND was achieved in selected patients with limited SLN metastases who were treated with breast conservation surgery and whole breast irradiation [4, 5, 25] . It was also shown in clinical practice that nodal extent does not appear to affect the number of patients receiving adjuvant chemotherapy [27] . However, it is accepted that in patients with extended LN involvement, adjuvant chemotherapy was still considered as necessary and that the extent of nodal involvement represents a factor for inclusion of chemotherapy in systemic therapy [17, 18, 28, 29] . Patients with HER2+ early breast cancer still depend on lymph node status for selection of the most appropriate systemic therapy. Nodal status is important for the choice of the chemotherapy regimen [30] or the adjuvant indication for treating patients with dual HER2 blockade using pertuzumab to trastuzumab and chemotherapy [31] . Furthermore, for patients with ER+/HER2- early breast cancer node-positive status is crucial for adding chemotherapy to endocrine treatment and for the duration of endocrine therapy [29] . Thus, accurate diagnosis of the axilla nodal stage is still important because it offers the possibility for choosing the most appropriate treatment regimens for each individual patient. Furthermore, a negative clinical nodal status does not definitively exclude axillary disease, as our study confirms that about 26% of patients with a clinical node-negative breast cancer had metastases to the axillary nodes. SLN evaluation is the reference standard for determining nodal involvement and should be offered to patients with a preoperative negative axilla assessment [1–3, 32, 33] .

Indisputably, there is currently a cohort of patients with positive SLNs who require an additional ALND. One reason for this is the fact that the final number of positive lymph nodes (only one LN+, two or three LN+ or more than 4 LN+) determines the accurate systemic treatment and radiotherapy. Another reason is that a group of these patients has a benefit in loco-regional control and survival [34, 35] . In the event that no intraoperative evaluation of the SLNs is offered to these patients, they will run the risk of a second surgical intervention with the same risk of complications as after an immediate ALND. Pathological assessment and morbidity after a delayed ALND were not significantly different from the immediate ALND [26] . These findings demonstrate that there is no disadvantage of an immediate ALND. Nevertheless, delayed ALND and a second surgical intervention cause considerable unnecessary costs [22, 36] as well as emotional distress for the patient, increased risk of infection and complications from anesthesia associated with the additional surgical procedures.

## Conclusions

The results of the present study clearly demonstrate that TIC as a screening system and subsequent FS of cytologically-positive SLNs is a highly reliable procedure for detecting SLN macrometastases that preserves the tissue from intraoperative negative SLN for the final histological diagnosis. Our comprehensive data demonstrate that this intraoperative procedure provides an outstanding evaluation of patients with more aggressive tumors, such as luminal-B, HER-2 positive or triple negative breast cancer as well as patients with highly proliferating tumors. Specifically, it allows accurate identification of patients with a high risk of extended axillary involvement directly during surgery, and has no disadvantages for patients without SLN metastases. Thus, the method represents an effective intervention that avoids unnecessary costs and, most importantly, can protect patients from the risks and inconveniences associated with a second surgical intervention.

## Data Availability

The cytological specimens, frozen sections and paraffin-embedded slides as well as paraffin-embedded tumors are archived in the Institute of Pathology at the University Medical Center in Mainz.

## Abbreviations

SLN: Sentinal lymph node
LN: Lymph node
ALND: Axillary lymph node dissection
TIC: Touch imprint cytology
FS: Frozen section
ME: Method error
SE: Sampling error
MI: Misinterpretation
TP: True positive
FN: False negative
TN: True negative
FP: False positive
PPV: Positive predictive value
NPV: Negative predictive value
